# Supplementary figures and images for: Stem cell proliferation and differentiation during larval metamorphosis of the model tapeworm Hymenolepis microstoma
Source: Front Cell Infect Microbiol. 2023 Oct 16;13:1286190. doi: 10.3389/fcimb.2023.1286190 (PMC10614006; doi:10.3389/fcimb.2023.1286190)

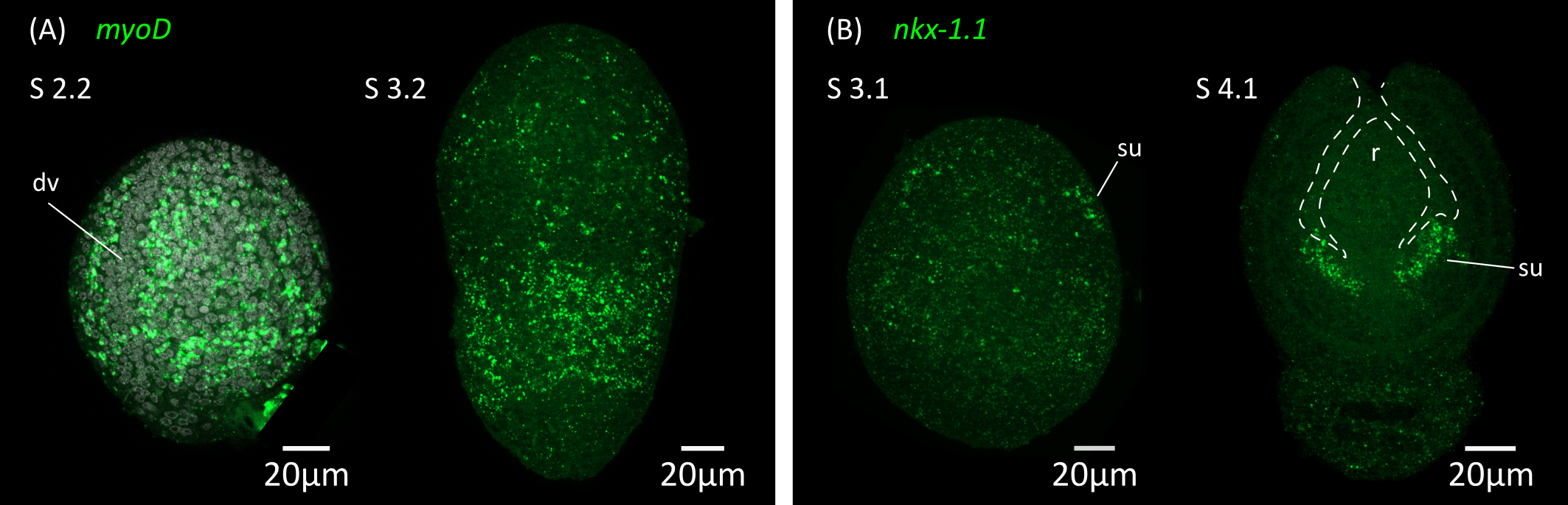

Supplement: Supplementary Figure 1 — Expression of myoD (A) and nkx-1.1 (B) homologs during larval metamorphosis. dv, lateral band of marginal myocitons; su, sucker primordia/developing sucker. [file Image_1.tiff]
